# Microstructure determines crystallinity-driven singlet fission efficiency in diF-TES-ADT

**DOI:** 10.1038/s41598-025-08427-y

**Published:** 2025-07-03

**Authors:** Hoyeon Choi, Stefan Skalsky, David G. Bossanyi, Jenny Clark, Patrick Parkinson

**Affiliations:** 1 Department of Physics and Astronomy and the Photon Science Institute, University of Manchester, Oxford Road, M13 9PL Manchester, UK; 2 School of Mathematical and Physical Sciences, The University of Sheffield, Hounsfield Road, S3 7RH Sheffield, UK; 3 Department of Physics, University of Bath, Claverton Down, BA2 7AY Bath, UK

**Keywords:** Optical materials and structures, Optical techniques, Solar cells, Microscopy

## Abstract

Singlet fission (SF) describes the conversion of a single photon-generated excited state into two triplet excitons through an initial singlet state. Despite its significance for solar energy applications, the relationship between microstructure, temperature, and SF efficiency remains poorly understood. Using cryogenic fluorescence microscopy, we correlate primary singlet fission (PSF) efficiency with local film morphology in a prototypical high-efficiency anthradithiophene (diF-TES-ADT) thin film. Our hyperspectral microscopy measurements of absorption and emission with sub-micron resolution reveal spatially inhomogeneous PSF efficiency that correlates directly with local crystallinity. Temperature- and time-resolved spectroscopy demonstrate that enhanced PSF efficiency in highly crystalline regions results from favorable endothermic alignment of a charge-transfer (CT) state. These findings emphasize how spatial inhomogeneity critically impacts SF film performance and caution against relying solely on spatially averaged metrics when evaluating SF materials.

## Introduction

The achievable power conversion performance of photovoltaic devices is fundamentally constrained by the Shockley-Queisser (SQ) limit, where the maximum efficiency for a single junction cell is primarily determined by the bandgap of the absorbing material^1–11^. One promising strategy to overcome this limit is singlet fission (SF), where a single photon absorption generates a pair of triplet excitons^6–8,11^, offering advantages similar to multiple exciton generation. Numerous photophysical studies have investigated the SF mechanism to improve its efficiency and optimize molecular design and fabrication conditions^4,12,13^. The evolution of triplet pairs ($2\times T_1$) from a singlet ($S_1$ ) proceeds through several intermediate steps^3,6,8,14^:

1 $$\begin{aligned} |{S_1}\rangle \rightarrow |{^1(TT)}\rangle \rightarrow |{(T_1...T_1)^l}\rangle \rightarrow 2\times |{T_1}\rangle \end{aligned}$$

The first process, primary singlet fission (PSF), converts the photogenerated singlet exciton ($S_1$ ) into a correlated triplet pair state ($^1(TT)$ ) with preserved spin character (overall spin-0) through an ultrafast process^5,6,8,14^. This correlated triplet state then evolves into electronically independent but spin-correlated triplet-pairs ($(T_1...T_1)^l$) on a longer timescale. Finally, spin-decoherence produces a pair of dissociated (spin-1) $T_1$ states that can be harvested^6,8,9,14^. A detailed model to describe this evolution was initially established by Merrifield^15^, and has been expanded by Bossanyi and colleagues^8^.

Molecular packing significantly influences the physical properties of organic molecules, particularly their charge transfer mechanisms and consequently, the SF process^2,4,5,9,10,16–18^. The impact of local nano- and microscale environments on SF photophysical dynamics reveals complex behavior arising from cascading inter- and intramolecular transitions^4,5,9,10,13^, each dependent on nuclear motion^4,5,9,16,17^. In an endothermic SF material ($E_{S_1}<2\times E_{T_1}$), fission has been shown to proceed *via* a charge transfer (CT) state to lower the thermal activation barrier ($E_b = 2E_{T_1}-E_{S_1} > 0$).

The PSF rate becomes particularly sensitive to the local environment when mediated by this intermediate CT state, as it couples to environmental vibronic modes^5,10,19^. Local vibrational perturbations thus determine overall SF efficiency, with PSF competing against unwanted pathways that depopulate the $^1(TT)$ state^9,10,12,20^. Recent experiments have demonstrated environmental control through solvent-based methods: tetracene and anthradithiopene (ADT) derivatives show SF efficiency correlating with solvent polarity^20^. Additionally, side-chain modifications in ADT derivatives can control molecular packing, enabling more favorable energy level alignment in specific morphologies to enhance both exciton transport and charge transfer properties^5,10,12,21^. While highly-controlled deposition methods continue to advance, spin-coating remains prevalent due to its simplicity and potential for economical mass production. However, the relationship between local morphology and SF efficiency in spin-coated endothermic SF thin layers remains a subject of debate, particularly compared to well-studied single crystal or solution systems that are less practical for device applications^5,18,20^.

ADT and its derivatives are widely studied as endothermic SF materials, as they enable singlet fission through either thermal activation or fast vibrationally-mediated processes to overcome the energy barrier between $S_1$ and $2 \times T_1$^5^. Among these compounds, diF-TES-ADT (2,8-difluoro-5,11-bis(triethylsilylethynyl)anthradithiophene) exhibits superior SF efficiency due to its characteristic brickwork structure^5,12^. The compound’s emissive $^1(TT)$ state at specific temperatures, combined with a relatively low density of excimer states barely detectable at the low temperature, makes it an ideal platform for studying morphology-dependent exciton dynamics. This distinction is particularly valuable since excimer states, which can act as unfavorable charge traps that compete with the SF process^5,8^. Therefore, diF-TES-ADT films provides an ideal platform for observing the population of $^1(TT)$ via Herzberg-Teller intensity borrowing under controlled temperature conditions, independently of loss pathways due to the activation of excimer^8^. In particular, polymorphic films composed of diF-TES-ADT reveal the variations in SF efficiency correlated to the localized phase heterogeneity inherent to the spin-coating process. This holistic insight provides a valuable guideline for future optimization of spin-coating methods, and molecular design strategies.

Conventional spot-to-spot measurements system are hindered by several critical limitations, including mechanical vibrations, protracted acquisition times and challenges in studying the same regions in both transmission and absorption. These factors collectively extend the total measurement duration required to obtain statistically significant data, while introducing temporal variations that can confound the interpretation of intrinsic local physical properties. In contrast, our hyperspectral imaging system provides rapid, large-area spatial mapping under stable and uniform conditions, making a robust measurement of the degree of correlation between separately measured physical parameters possible across a large spatial scale.

In this study, we demonstrate how local morphology influences SF efficiency in spin-coated diF-TES-ADT through temperature-dependent hyperspectral imaging. Our all-optical characterization system visualizes local SF efficiencies across varying molecular crystallinity, revealing efficiency fluctuations of $\sim$70%. Notably, these efficiency variations persist below the critical temperature where endothermic SF typically becomes thermodynamically unfavorable, suggesting that molecular crystallinity directly modulates the SF energy barrier. Micrometre-scale ultrafast pump-probe measurements further establish this connection between spatial heterogeneity and molecular crystallinity. These measurements confirm that a CT state accelerates PSF, effectively competing with excimer-related loss pathways.

## Results

**Fig. 1:**
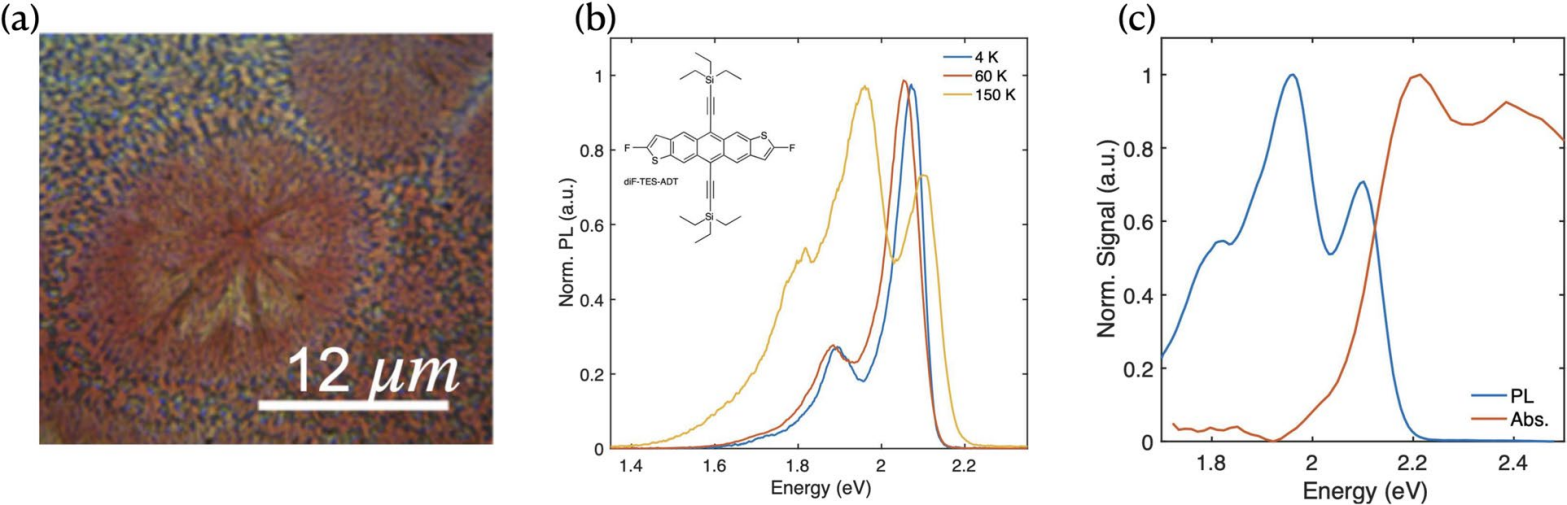
(**a**) An optical-transmission microscopy image of the diF-TES-ADT thin film. (**b**) A PL spectrum with an excitation spot area of 75 $\mu$m$^2$ at different sample temperatures of 4, 60 and 150 K; the inset shows the molecular structure of the diF-TES-ADT . (**c**) The normalized PL and absorption spectrum averaged over 700 $\mu$m$^2$ at 150 K, obtained using calibrated hyperspectral photoluminescence microscopy.

Figure 1a shows a bright-field optical microscopic image of a polycrystalline diF-TES-ADT film spin-coated on a z-cut quartz substrate. The spin-coating process yielded a relatively uniform flat surface with an average thickness of 134 nm and a root mean square roughness of 3 nm (see Fig. S1a Supporting Information). The film also exhibits distinct micro-morphological domains ranging from several $\mu$m to tens of $\mu$m in size, representing local variations in crystallization. These domains display lower optical contrast than their surrounding regions, likely due to reduced optical scattering at their flattened surfaces compared with the more randomly oriented microcrystalline areas (Fig. S1b,c Supporting Information).

Temperature-dependent steady-state photoluminescence (PL) spectra were measured using a 405 nm excitation wavelength at a photon density of $1.3 \times 10^{17}$ photons/s$\cdot$cm$^2$ (63 mW/cm$^2$). Measurements were performed at a fixed location using a large excitation and detection spot to average over morphological features.

The PL spectrum below 100 K (Fig. 1b) exhibits two peaks at 2.07 eV and 1.89 eV (measured at 4 K), corresponding to the 0-0 and 0-1 transitions of $S_1$ . These peaks redshift by 6 meV as temperature increases from 4 K to 60 K, consistent with the change in thermal energy ($k_B\Delta T = 6.5$ meV). At low temperature, we observe no signature of singlet fission, which would manifest as quenching of $S_1$ emission accompanied by emergence of $^1(TT)$ associated emission peaks^6,8^.

While triplet emission is typically spin-forbidden, the Herzberg-Teller mechanism enables hybridization between optically dark $^1(TT)$ and emissive $S_1$ states, creating bright $^1(TT)$ states accessible through TTA^5,8,22^. Above a critical temperature of 100 K, the steady-state emission exhibits both prompt $S_1$ emission and $^1(TT)$ emission, the latter resulting from sequential SF and TTA^6,8,22^.

The emission from $^1(TT)$ occurs at $2 \times E_{T_1} - \hbar \omega _{vib}$, where $\hbar \omega _{vib}$ is the vibrational quantum of C=C bonding, making it distinguishable from $S_1$ emission^8^. At 150 K, the steady-state PL spectrum of diF-TES-ADT shows three distinct peaks at 2.10, 1.96, and 1.85 eV, with $E_{T_1} = 1.08$ eV and $\hbar \omega _{vib} = 0.18$ eV^8^. The highest-energy peak at 2.10 eV corresponds to the 0-0 emission of $S_1$ , while the two lower-energy peaks arise from $^1(TT)$ ^6,8^. diF-TES-ADT crystallizes in a simple brickwork structure that promotes H-aggregation, with no reported phase transitions in the measured temperature range^6,8^. Time-resolved spectroscopic studies have conclusively demonstrated that these spectral features originate from the singlet state and the correlated triplet pair following singlet fission^6,8^.

### Wide-field absorption

We perform microabsorption spectroscopy using white-light back-illumination to gain insight into crystal properties of the film such as local Stokes shift. In Fig. 1c, the area-averaged absorption (over a region of 700 $\mu$m$^2$) shows the $S_0 \rightarrow S_1$ transition ($A_{0-0}$) at 2.224 eV. The area-averaged Stokes shift ($E_\textrm{Stokes} = E_{A_{0-0}} - E_{S_1}$) is 0.126 eV, providing an estimate for the singlet Frenkel energy $E_\textrm{Frenkel} = E_{A_{0-0}} - \frac{1}{2}E_\textrm{Stokes} = 2.16$ eV^12,23^. The energy barrier in this weakly endothermic system is given by $E_b = E_\textrm{Frenkel} - 2E_{T_1} \approx 0$ eV, where $E_{T_1} = 1.08$ eV^12^. The barrier is thus estimated to be smaller than 10 meV, which is below the thermal energy at $T = 150$ K ($k_\textrm{B}T = 12.9$ meV), although it is noted that this is a crude measure due to the inherent breadth in a Boltzmann distribution. At $T = 60$ K, we observe $E_{A_{0-0}} = 2.212$ eV and a Stokes shift of 0.138 eV, leading to an energy barrier of 16 meV – notably exceeding the thermal energy at this temperature^4,8^. A map of Stokes shift at room temperature is provided in Supporting Information Fig. S5. The temperature-dependent behavior of diF-TES-ADT emission directly correlates with this internal endothermic barrier, explaining the absence of $^1(TT)$ -related emission below 100 K^6^.

At 150 K, the 1.96 eV signal primarily originates from $^1(TT)$ , with residual contributions from the $S_1$ 0-1 progression^4,6,8^. At 4 K, the relative intensity ratio of 0-0 and 0-1 emission ($I_{0-0}/I_{0-1}$) shows minimal dependence on excitation power (1.05 at lowest power, 0.97 at highest $\Pi _{ex}$, see Supporting Information Fig. S3). This indicates that at 150 K, variations in the 1.96 eV signal relative to the 2.10 eV peak reflect changes in $^1(TT)$ density and local SF efficiency rather than local exciton density (see Supporting Information Fig. S2). We therefore define the SF efficiency parameter as $\eta _{SF} = \int I[^1(TT)]/\int I[S_1]$ for subsequent analysis.

### Micro-spectroscopy mapping

Although area-averaged spectroscopy is commonly used to study SF processes, morphological variations at sub-micron length scales play a determining role in excitonic dynamics. We employed a hyperspectral imaging system (using a tunable filter approach described in the methods section) to generate calibrated three-dimensional datasets in space and photon energy for both absorption and emission. By fitting a model to each pixel, we extracted emission parameters ($S_1$ energy, intensity, and width; $^1(TT)$ energy, intensity, and width) and absorption parameters (amplitude, onset position, and gradient). These parameters enabled calculation of the SF efficiency $\eta _{SF}$.

**Fig. 2:**
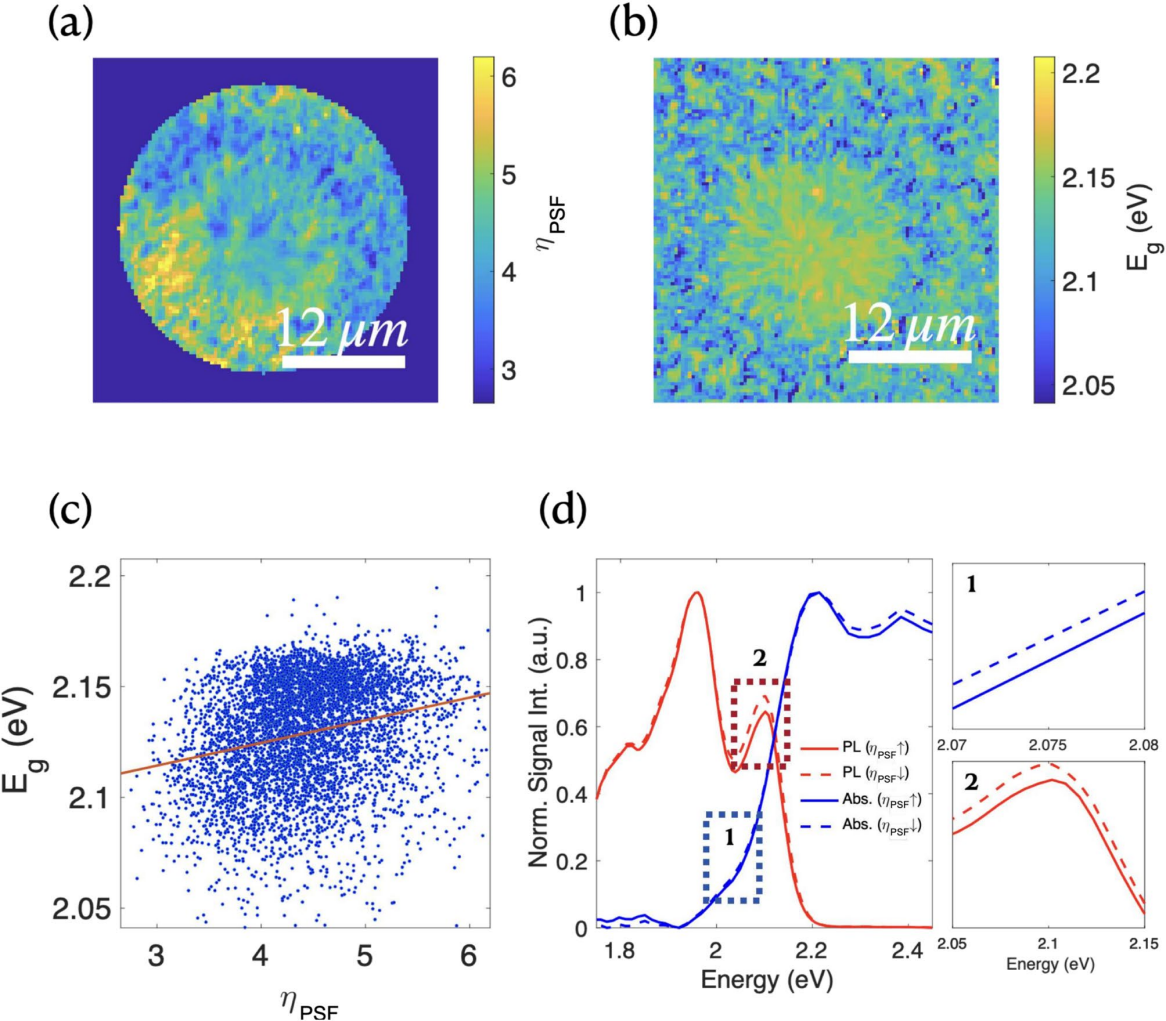
Processed PL and absorption maps at 150 K showing (**a**) the variance of $\eta _{SF}$ and (**b**) the absorption edge ($E_g$). (**c**) The correlation between $\eta _{SF}$ and $E_g$. (**d**) (Left) the average PL and absorption of high and low $\eta _{SF}$ regions, with close up detail of (d1) the absorption edge and (d2) the $S_1$ peaks as marked.

Figure 2a shows a spatial map of local SF efficiency at 150 K for the region shown in Fig. 1a, revealing variations in $\eta _{SF}$ by a factor of 2.39. These local variations correlate with morphological features observed through optical microscopy. Figure 2b demonstrates distinctive absorption patterns associated with these morphological features, particularly showing higher absorption edge energies ($E_{g}$) in extended crystalline regions (further detail in Supporting Information Fig. S4a,b).

Figure 2c reveals a significant positive correlation between $\eta _{SF}$ and absorption edge energy ($E_{g}$), with a Pearson’s linear correlation coefficient of $r = 0.229$ ($p < 1 \times 10^{-6}$). Figure 2d compares the average PL and absorption spectra for regions with the highest 5% and lowest 5% $\eta _{SF}$ values. Regions with higher $\eta _{SF}$ exhibit $S_1$ emission quenching due to SF, accompanied by a sharper absorption edge ($\nabla A_{0-0}$).

#### Photoluminescence modelling

Below 90 K, thermal energy is insufficient to overcome the energetic barrier ($2\times E_{T_1} - E_{S_1} < 0$)^6^. At this temperature, the PL spectrum consists solely of the Franck-Condon (F-C) progression of $S_1$ . This allows us to study the vibronic structure without singlet fission effects, as shown in Fig. S4c)^5,6,8,12^. The modified F-C progression model considering a variable 0-0 amplitude follows that proposed by Spano and Silva^24^,

2 $$\begin{aligned} I(\omega ) \propto (\hbar \omega )^3n(\omega )\left[ \alpha \Gamma (E_0,L)+\sum _{m=1}\frac{S^m}{m!}\Gamma (E_0-mE_{ph},\sigma )\right] . \end{aligned}$$

In this expression, $n(\omega )$ represents the real part of the refractive index at optical frequency $\omega$, *m* enumerates the vibrational level and *S* is the Huang-Rhys factor quantifying the coupling between electronic transitions and lattice vibrations. The energy of the 0-0 emission is given by $E_0$, while $E_{ph}$ represents the phonon energy of the C=C symmetric stretch (0.18 eV). The line shape function $\Gamma$ is approximated as Gaussian, with width $\sigma$ for the vibronic transitions and *L* for the 0–0 transition, where *L* is the Gaussian linewidth associated with the 0–0 peak at low temperature. At low temperature (4 K in our system), the dimensionless factor $\alpha$, which depends on disorder width and spatial correlation length, takes values of 0.38–0.4. Following excitation, the exciton relaxes towards a high symmetry-ordered state^5^. The aggregation state of diF-TES-ADT can be determined by fitting the steady-state PL spectrum to this $I(\omega )$ model.

diF-TES-ADT molecules form H-aggregates, which quench the 0-0 transition relative to the 0-1 emission. The ratio $I_{0-1}/I_{0-0}$ serves as an established proxy for molecular crystallinity^23,25^. Figure S4a in the Supporting Information presents a spatial map of $I_{0-1}/I_{0-0}$, revealing inhomogeneous molecular packing throughout the polycrystalline diF-TES-ADT thin film. Regions of higher crystallinity exhibit higher absorption edge energies and correspondingly smaller $I_{0-0}$ values (Fig. S4d). In contrast, regions of low crystallinity display reduced absorption onset energies due to enhanced absorption tails characteristic of molecular disorder^4,23^. Since the 0-1 singlet peak becomes indistinguishable above 100 K and direct spatial correlation between low- and high-temperature measurements is impractical in our experimental arrangement, we utilize the absorption edge energy as a reliable proxy for crystallinity.

**Fig. 3:**
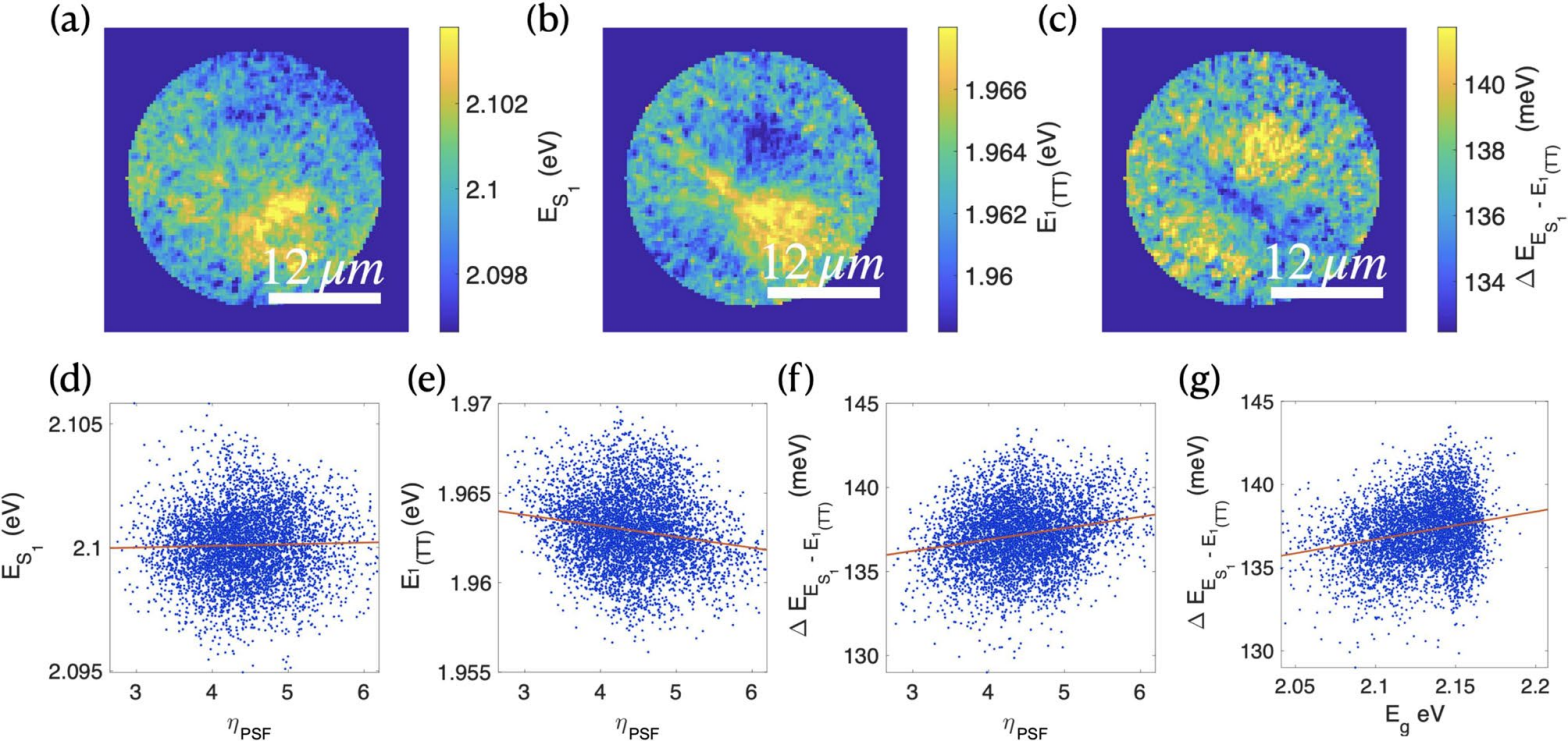
Processed PL maps obtained at 150 K of (**a**) energy variance of $E_{S_1}$ and (**b**) $E_{^1(TT)}$, and (**c**) the energy difference between $S_1$ and $^1(TT)$ : $\Delta E_{S_1 - ^1(TT)}$. Correlation between $\eta _{SF}$ and (**d**) $E_{^1(TT)}$, (**e**) $E_{S_1}$ and (**f**) $\Delta E_{S_1 - ^1(TT)}$. (**g**) shows the correlation between $E_{g}$ and $\Delta E_{S_1 - ^1(TT)}$.

Figure 3a–c reveals distinct spatial distributions of $S_1$ (0-0) and $^1(TT)$ emission energies, and in the difference between these energies. Both transitions vary across the diF-TES-ADT film, with spatial mapping indicating dependencies on local morphology and crystallinity. This variation aligns with previous studies showing that the $^1(TT)$ state rapidly acquires vibronic energy within picoseconds of excitation to achieve optimal fission geometries, making it sensitive to local molecular packing^4,9,20,26^.

To elucidate the thermodynamic drivers of local SF variations, we analyzed correlations between SF efficiency ($\eta _{SF}$) and transition energies (Fig. 3d–f). The $^1(TT)$ energy exhibits a significant negative correlation with efficiency ($r = -0.174$), while the $S_1$ transition energy shows minimal correlation ($r = 0.027$). These relationships suggest that strong vibronic coupling can simultaneously lower the $^1(TT)$ transition energy and enhance SF efficiency. Additionally, elevated $S_1$ state energies may facilitate overcoming the endothermic barrier to $T_1$ formation. The energy difference between these levels demonstrates the strongest correlation with SF efficiency ($r = 0.206$), and further correlates with the absorption edge ($E_g$, $r = 0.227$) as shown in Fig. 3g.

### Time-resolved photoluminescence

The dynamics between photon absorption and triplet formation span multiple timescales^4,7,10^. We performed time- and spectrally-resolved emission measurements in regions of high and low crystallinity (shown in Supporting Information Fig. S6a–d,f,g at two temperatures: 122 and 60 K, which lie just above and below the critical temperature for thermally enabled SF, respectively^8^. While SF is most efficient at 150 K, the significantly reduced $S_1$ signal intensity at this temperature complicates measurements. Therefore, we conducted our experiments at 122 K, where we could still clearly observe the signature of SF processes (Fig. S6e,h).

**Fig. 4:**
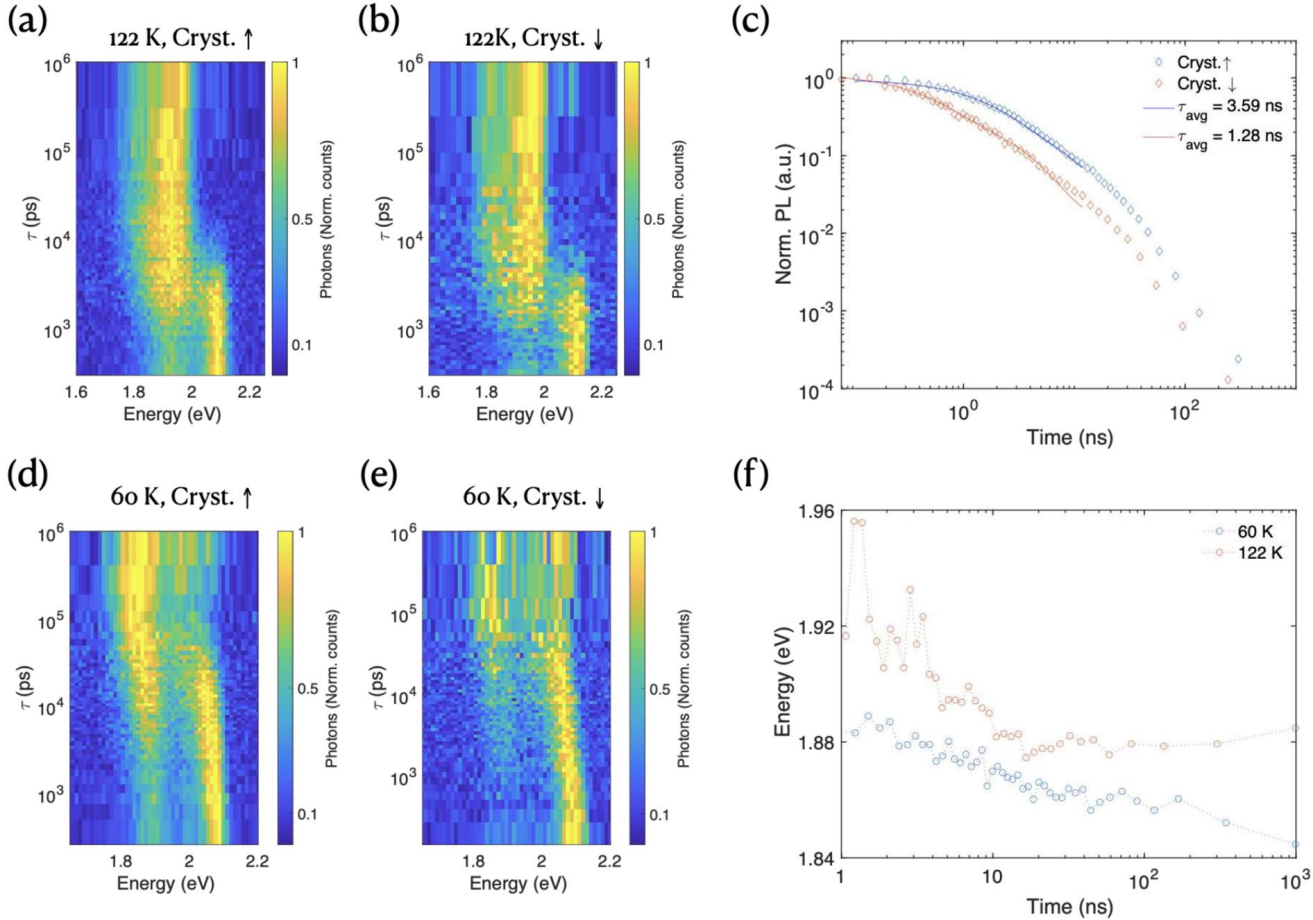
Time resolved PL spectroscopy, showing high and low temperature (**a**,**d**) the time resolved PL at 122 K (60 K) in the high crystalline region and (**b**,**e**) low crystalline regions. (**c**) The $^1(TT)$ emission lifetime in high/low crystalline regions. (**f**) The evolution of the $^1(TT)$ energy as a function of time for different temperatures.

Figure 4a,b show the normalized temporal evolution of PL in highly crystalline and poorly crystalline regions of the film, respectively (selection criteria detailed in the Supporting Information). At 122 K, both regions exhibit conversion from $S_1$ to $^1(TT)$ emission within $\tau _{S_1} < 5$ ns after excitation, consistent with previous ensemble measurements^8^. The $^1(TT)$ evolution in Fig. 4c reveals distinct lifetimes: 3.59 ns in the highly crystalline region versus 1.28 ns in the low crystallinity region. Since PSF dominates $^1(TT)$ population at early times ($\tau < 100$ ns), this extended emission indicates a slower $|S_1\rangle \rightarrow |^1(TT)\rangle$ transition. These dynamics remain consistent across our measured excitation power range (see Supporting Information, Fig. S6e)^8^.

At 4 K, no SF is expected or observed. The $S_1$ emission energy redshifts over 100 ns after excitation ($\Delta E_{S_1, 4K} = 32\,$meV, Fig. S6f). At 60 K, this shift becomes more pronounced ($\Delta E_{S_1, 60K} = 71\,$meV). However, despite thermal energy remaining 5 meV below the critical temperature at 60 K, SF efficiency exhibits strong dependence on local morphology.

In highly crystalline regions (Fig. 4d,e), emission associated with the $^1(TT)$ state emerges approximately 10 ns after excitation. In contrast, poorly crystalline regions show no clear SF signature. This morphology-dependent behavior suggests an endothermic energy barrier that can be overcome through a CT-mediated pathway. Two primary factors govern efficient PSF: thermal energy, which increases phonon population to assist to overcome the endothermic barrier; and local morphology, which can stabilize CT-like intermediate configurations, thereby enabling CT-mediated PSF even when thermal activation alone is insufficient to reach the fission threshold^12^. Time-resolved PL measurements enable tracking of the peak emission energy, quantifying the dominant emission process as a function of time (Fig. 4f). At 122 K, the rapid decrease in emission energy within the first 20 ns after excitation indicates that the majority of PSF occurs within this timeframe.

### Micro-transient absorption spectroscopy

At room temperature, PSF occurs on a sub-picosecond timescale^7,8^. To correlate these ultrafast dynamics with structure, we employed room temperature micro-transient absorption spectroscopy. The morphological features ($\sim$10 $\mu$m) in our films exceed the diameter of both pump ($\sim$6 $\mu$m) and probe ($\sim$4 $\mu$m) beams, enabling us to selectively target specific domains for direct comparison between regions with and without such features. To complement this, we used *in-situ* quasi-steady-state PL measurements, recorded simultaneously with transient absorption signals (Fig. S7a, allowing us to track emissive $^1(TT)$ intensity independently of excimer formation, which does not contribute to emissive $^1(TT)$^8^. Previous studies of diF-TES-ADT identified a broad negative signal centered at 1.66 eV extending to 1.95 eV as photo-induced absorption (PIA) of the initial singlet state ($|S_1\rangle \rightarrow |S_n\rangle$)^5,6,12^. While outside our spectral window, PIA of the emissive $^1(TT)$ state ($|^1(TT)\rangle \rightarrow |S_2\rangle$) was previously observed at 1.26 eV^5,6,12^, with the ground state bleach (GSB) of $|S_0\rangle \rightarrow |S_1\rangle$ transition at 2.3 eV.

**Fig. 5:**
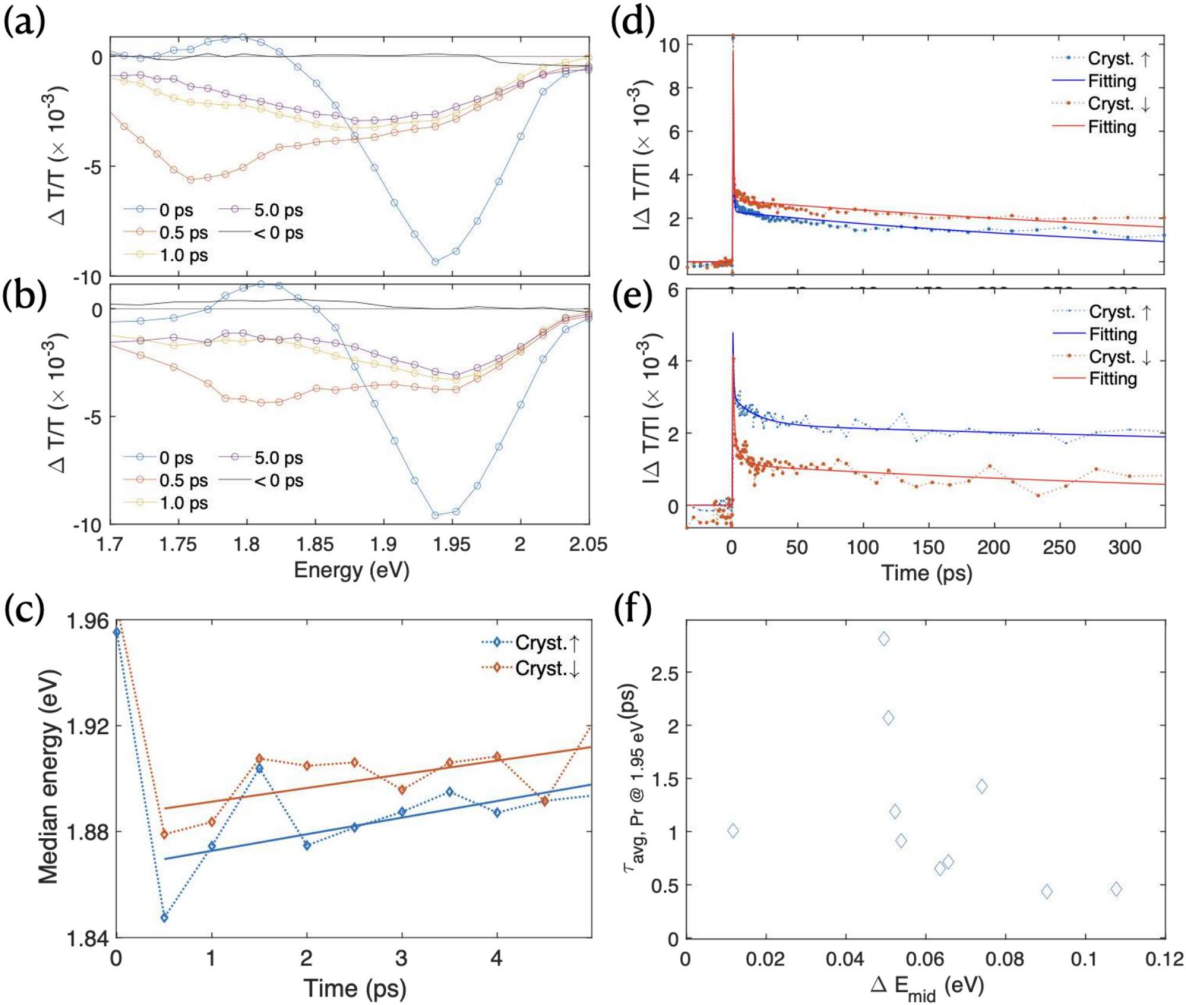
Micro-transient absorption on selected regions associated with high and low crystallinity regions (identified using PL). (**a**) The evolution of $\Delta T/T$ in the high crystalline and (**b**) the poor crystalline regions. (**c**) The evolution of the median energy ($E_{mid}$) of $\Delta T/T$ at early times. (**d**) The $\Delta T/T$ probed at 1.95 eV, (**e**) and at 1.85 eV. (**f**) The correlation between $E_{mid}$ and $\tau _{avg,1.95\textrm{eV}}$ measured at 10 points in the film.

Figure 5a,b shows spectral kinetics from high and low crystalline regions at 0, 0.5, 1, and 5 ps, with complete evolution data presented in Fig. S7b,c. Within 0.5 ps of excitation, $\Delta T/T$ exhibits a short-lived feature at 1.95 eV that relaxes on the sub-picosecond timescale in both crystalline regions. Additionally, we observe a positive $\Delta T/T$ signal at 1.81 eV. This stimulated emission (SE) signal has been attributed to the first excited state of an excimer occurring above 250 K^8,10,17,21,27^.

We use two identified spectral features to explore energy dynamics in the film: the singlet PIA at 1.95 eV and excimer SE at 1.81 eV. While these features exhibit spectral overlap, the signal at 1.95 eV primarily originates from $|{S_1}\rangle$, whereas at 1.80 eV both the SE of the excimer and PIA of $S_1$ contribute. To quantify the excimer emission proportion, we analyze the evolution of the median emission energy across 1.7 eV-2.05 eV. Fig. 5c shows that higher crystalline regions (with higher $\eta _{SF}$) exhibit larger median energy shifts at early times, before converging with lower crystalline regions after 5 ps.

This behavior is further evidenced in the dynamics at specific energies. The higher crystalline region shows faster decay at 1.95 eV (Fig. 5d) but slower decay at 1.85 eV (Fig. 5e). These kinetics, following ultrafast transfer between singlet and excimer states, suggest efficient carrier transfer into the $^1(TT)$ state. This interpretation aligns with time-resolved PL results (Fig. 4c), where the high crystalline region exhibits a 2.80$\times$ longer emission lifetime than the low crystalline region^4,10,17,20,27^.

Micro-transient absorption measurements provide additional insight into spatial variations of spectra and dynamics. Unlike our absorption and emission study (Figs. 2 and 3), direct imaging of crystallinity regions with this technique is challenging. Instead, we analyzed 10 randomly selected positions around a morphological feature (Fig. 5f). We compare the the median emission energy (indicating the rate of excimer energy transfer) with the recombination rate (indicating total singlet state energy transfer rate). While the correlation is not strong, the overall trend suggests that the faster singlet decay correlate with larger $S_1$ median energy shifts. This suggests that more rapid singlet decay may facilitate to more efficient charge transfer to the CT state by avoiding excimer state formation, where excimer population serves as an indicator of molecular crystallinity^10^.

## Conclusion

Our investigation of spin-coated diF-TES-ADT thin films reveals significant spatial variation in SF efficiency, with up to 2.39-fold differences at 150 K between regions of varying crystalline order. Through hyperspectral cryogenic absorption and photoluminescence imaging, we establish a direct correlation between local crystallinity and SF efficiency, supporting previous findings that CT state formation depends strongly on molecular crystal phase^12^. This combined spectroscopic approach enables reliable evaluation of crystallinity in disordered spin-coated films across controlled temperatures.

Time-resolved measurements from picoseconds to microseconds demonstrate that SF proceeds through both thermal and structural activation pathways. At room temperature, SF efficiency competes with an excimer-mediated loss channel, but enhanced crystalline ordering promotes transfer through the intermediate CT state. The observed spatial heterogeneity in SF efficiency underscores two critical insights: the fundamental role of crystallinity in enabling efficient SF, and the necessity of considering local structural variation when evaluating SF materials.

## Experimental methods

### Sample preparation

The diF-TES-ADT film was deposited on z-cut quartz by spin-coating at 1000 rpm at 60 s.

### Characterization

#### Hyperspectral image processing

The cryogenic hyperspectral imaging system is shown in Fig. S8a. A tunable liquid crystal spectral filter was used to provide spectral resolution, while an IR-enhanced CMOS camera was used for imaging. It is noted that the filter scrambled incoming polarization, rendering the measurements polarization independent. The system was calibrated using a spectrometer and blackbody source. Spatial resolution is obtained using an objective lens (Zeiss LD EC Epiplan-Neofluar 100x/0.90 DIC M27) integrated into a cryostat system. Photo-physical studies were carried out in variable temperatures(from $\sim$4 K to 300 K). A co-linear white light source was used for transmission measurements, while a de-focussed 405 nm excitation laser was used for photoluminescence study.

#### Interferometric time correlated single photon counting (iTCSPC)

A 405 nm pulsed diode laser at a frequency of 1 MHz and with pulse duration $\sim$80 ps was used to excite the sample using the cryo-microscopy described above. The samples were excited and emission collected in reflection configuration in a quasi-confocal arrangement. A optical fibre (core diameter of 100 $\mu$m) was used to transport the photoluminescence signal, which was was fed to our home-built interferometric TCSPC system previously described^28^ to provide time- and energy-resolved emission.

#### Micro transient absorption ($\mu$-TA)

Figure S8b shows the layout of $\mu$-TA. A primary laser pulse at 1030 nm, and 200 kHz with a pulse width of approximately 250 fs pumped an optical parametric amplifier to generate a 405 nm excitation pulse. This was used to excite the diF-TES-ADT thin film at room-temperature. The residual 1030 nm beam was used to generate white light by focusing onto sapphire plate. The pump and probe beams were aligned counter-propagating in a home-built microscope, with the broadband probe beam is focused by a reflective objective(40x, NA 0.5) to avoid chromatic aberration. The 405 nm pump beam is focused by the counter objective (20x, NA 0.45) with a pulse energy of 0.138 nJ over a $\sim 5$ *mu*m spot. The transmitted probe beam passes through a dichroic mirror (425 nm) to remove the contribution of the excitation beam and is fed into a spectrometer with a silicon avalanche photodiode (APD 130x, Thorlabs). A dark-field optical image of the sample is monitored using a CMOS camera. A motorized x-y stage enables large area scans with high spatial reproducibility. The obtained pump-probe signal is amplified by the digital signal processing through a synchronous analog digital converter which takes references from a pair of optical choppers for each of the pump and probe beam lines.

### Atomic force microscopy (AFM)

The AFM data was collected with a Multimode8 configured with a J scanner. The probe used was Scan Asyst air. The experimental mode used to obtain the line scanning in Figure S1 Supporting Information was Scan Asyst. The in-plane resolution is 97.65 nm per step.

## Supplementary Information

Supplementary Information.

## Data Availability

Data is provided within the manuscript and supplementary information. Raw datasets on transmission and absorption are available on Institutional Figshare (10.48420/28512701).

## References

[1] Busby, E. et al. A design strategy for intramolecular singlet fission mediated by charge-transfer states in donor-acceptor organic materials. *Nat. Mater.* **14**, 426–433 (2015). 25581625 10.1038/nmat4175

[2] Van Schenck, J., Mayonado, G., Anthony, J., Graham, M. & Ostroverkhova, O. Molecular packing-dependent exciton dynamics in functionalized anthradithiophene derivatives: From solutions to crystals. *J. Chem. Phys.* **153**, 164715 (2020). 33138416 10.1063/5.0026072

[3] Nandi, A., Manna, B. & Ghosh, R. Singlet fission dynamics in the 5, 12-bis (phenylethynyl) tetracene thin film. *J. Phys. Chem. C* **125**, 2583–2591 (2021).

[4] Stern, H. L. et al. Vibronically coherent ultrafast triplet-pair formation and subsequent thermally activated dissociation control efficient endothermic singlet fission. *Nat. Chem.* **9**, 1205–1212 (2017). 29168494 10.1038/nchem.2856

[5] Mayonado, G. et al. High-symmetry anthradithiophene molecular packing motifs promote thermally activated singlet fission. *J. Phys. Chem. C* **126**, 4433–4445 (2022).

[6] Yong, C. K. et al. The entangled triplet pair state in acene and heteroacene materials. *Nat. Commun.* **8**, 1–12 (2017). 28699637 10.1038/ncomms15953 PMC5510179

[7] Rao, A. & Friend, R. H. Harnessing singlet exciton fission to break the shockley-queisser limit. *Nat. Rev. Mater.* **2**, 1–12 (2017).

[8] Bossanyi, D. G. et al. Emissive spin-0 triplet-pairs are a direct product of triplet-triplet annihilation in pentacene single crystals and anthradithiophene films. *Nat. Chem.* **13**, 163–171 (2021). 33288892 10.1038/s41557-020-00593-y

[9] Seiler, H. et al. Nuclear dynamics of singlet exciton fission in pentacene single crystals. *Sci. Adv.* **7**, eabg0869 (2021). 34172443 10.1126/sciadv.abg0869 PMC8232917

[10] Dover, C. B. et al. Endothermic singlet fission is hindered by excimer formation. *Nat. Chem.* **10**, 305–310 (2018). 29461531 10.1038/nchem.2926

[11] Einzinger, M. et al. Sensitization of silicon by singlet exciton fission in tetracene. *Nature* **571**, 90–94 (2019). 31270480 10.1038/s41586-019-1339-4

[12] Mayonado, G., Vogt, K.T., Van Schenck, J.D., Ostroverkhova, O. & Graham, M.W. Packing morphology-dependent singlet fission in single crystal anthradithiophene derivatives. In *International Conference on Ultrafast Phenomena*, Th2A–4 (Optical Society of America, 2020).

[13] Huang, G. et al. Aggregation regulated ultrafast singlet fission pathways in tips-pentacene films. *Ultrafast Sci.* **4**, 0057 (2024).

[14] Lee, T. S. et al. Triplet energy transfer governs the dissociation of the correlated triplet pair in exothermic singlet fission. *J. Phys. Chem. Lett.* **9**, 4087–4095 (2018). 29976063 10.1021/acs.jpclett.8b01834

[15] Merrifield, R., Avakian, P. & Groff, R. Fission of singlet excitons into pairs of triplet excitons in tetracene crystals. *Chem. Phys. Lett.* **3**, 386–388 (1969).

[16] Duan, H.-G. et al. Intermolecular vibrations mediate ultrafast singlet fission. *Sci. Adv.* **6**, eabb0052 (2020). 32948583 10.1126/sciadv.abb0052 PMC7500928

[17] Piland, G. B. & Bardeen, C. J. How morphology affects singlet fission in crystalline tetracene. *J. Phys. Chem. Lett.* **6**, 1841–1846 (2015). 26263258 10.1021/acs.jpclett.5b00569

[18] Volek, T. S. et al. Structural disorder at the edges of rubrene crystals enhances singlet fission. *J. Phys. Chem. Lett.* **14**, 11497–11505 (2023). 38088867 10.1021/acs.jpclett.3c02845

[19] Kim, H. & Zimmerman, P. M. Coupled double triplet state in singlet fission. *Phys. Chem. Chem. Phys.* **20**, 30083–30094 (2018). 30484452 10.1039/c8cp06256k

[20] Mardazad, S. et al. Quantum dynamics simulation of intramolecular singlet fission in covalently linked tetracene dimer. *J. Chem. Phys.* **155**, 194101 (2021). 34800955 10.1063/5.0068292

[21] Hoffman, B. C. et al. Intrinsic charge trapping observed as surface potential variations in dif-tes-adt films. *ACS Appl. Mater. Interfaces* **8**, 21490–21496 (2016). 27466823 10.1021/acsami.6b03886

[22] Musser, A. J. & Clark, J. Triplet-pair states in organic semiconductors. *Annu. Rev. Phys. Chem.* **70**, 323–351 (2019). 31174458 10.1146/annurev-physchem-042018-052435

[23] Gierschner, J. et al. Luminescence in crystalline organic materials: from molecules to molecular solids. *Adv. Opt. Mater.* **9**, 2002251 (2021).

[24] Spano, F. C. & Silva, C. H-and j-aggregate behavior in polymeric semiconductors. *Annu. Rev. Phys. Chem.* **65**, 477–500 (2014). 24423378 10.1146/annurev-physchem-040513-103639

[25] Clark, J., Silva, C., Friend, R. H. & Spano, F. C. Role of intermolecular coupling in the photophysics of disordered organic semiconductors: aggregate emission in regioregular polythiophene. *Phys. Rev. Lett.* **98**, 206406 (2007). 17677723 10.1103/PhysRevLett.98.206406

[26] Jones, A. C., Kearns, N. M., Ho, J.-J., Flach, J. T. & Zanni, M. T. Impact of non-equilibrium molecular packings on singlet fission in microcrystals observed using 2d white-light microscopy. *Nat. Chem.* **12**, 40–47 (2020). 31792384 10.1038/s41557-019-0368-9

[27] Hausch, J. et al. Distinguishing between triplet-pair state and excimer emission in singlet fission chromophores using mixed thin films. *J. Phys. Chem. C* **126**, 6686–6693 (2022).

[28] Skalsky, S. et al. Heterostructure and q-factor engineering for low-threshold and persistent nanowire lasing. *Light Sci. Appl.* **9**, 1–10 (2020). 32194957 10.1038/s41377-020-0279-y PMC7078256

