# Supplementary material for: Microstructure determines crystallinity-driven singlet fission efficiency in diF-TES-ADT
Source: Sci Rep. 2025 Jul 3;15:23737. doi: 10.1038/s41598-025-08427-y (PMC12229493; doi:10.1038/s41598-025-08427-y)
Supplement: Supplementary file 1 — Supplementary Information. [file 41598_2025_8427_MOESM1_ESM.pdf]

Supporting Information: Microstructure Determines Crystallinity-Driven Singlet Fission Efficiency in diF-TES-ADT

Hoyeon Choi,[*,†,¶] Stefan Skalsky,[†] David G. Bossanyi,[‡] Jenny Clark,[‡] and Patrick Parkinson[*,†]

†*Department of Physics and Astronomy, The University of Manchester, Oxford Road, Manchester, M13 9PL, United Kingdom*

‡*Department of Physics and Astronomy, The University of Sheffield, Hounsfield Road, Sheffield, S3 7RH, United Kingdom*

¶*Current Address: Department of Physics, University of Bath, Claverton Down, Bath, BA2 7AY, United Kingdom*

E-mail: hc2403@bath.ac.uk; patrick.parkinson@manchester.ac.uk

Thickness characterization

The sample thickness was assessed using atomic force microscopy (AFM) as described in the manuscript. The film thickness was measured to be 134 nm as shown in Figure S1a with an root-mean-squared roughness of 3 nm. The variation in film absorption was more significant, with a bimodal distribution observed (Figure S1b). We segmented the absorption plots into two regions, as shown in Figure S1c, for our analysis in the main text.

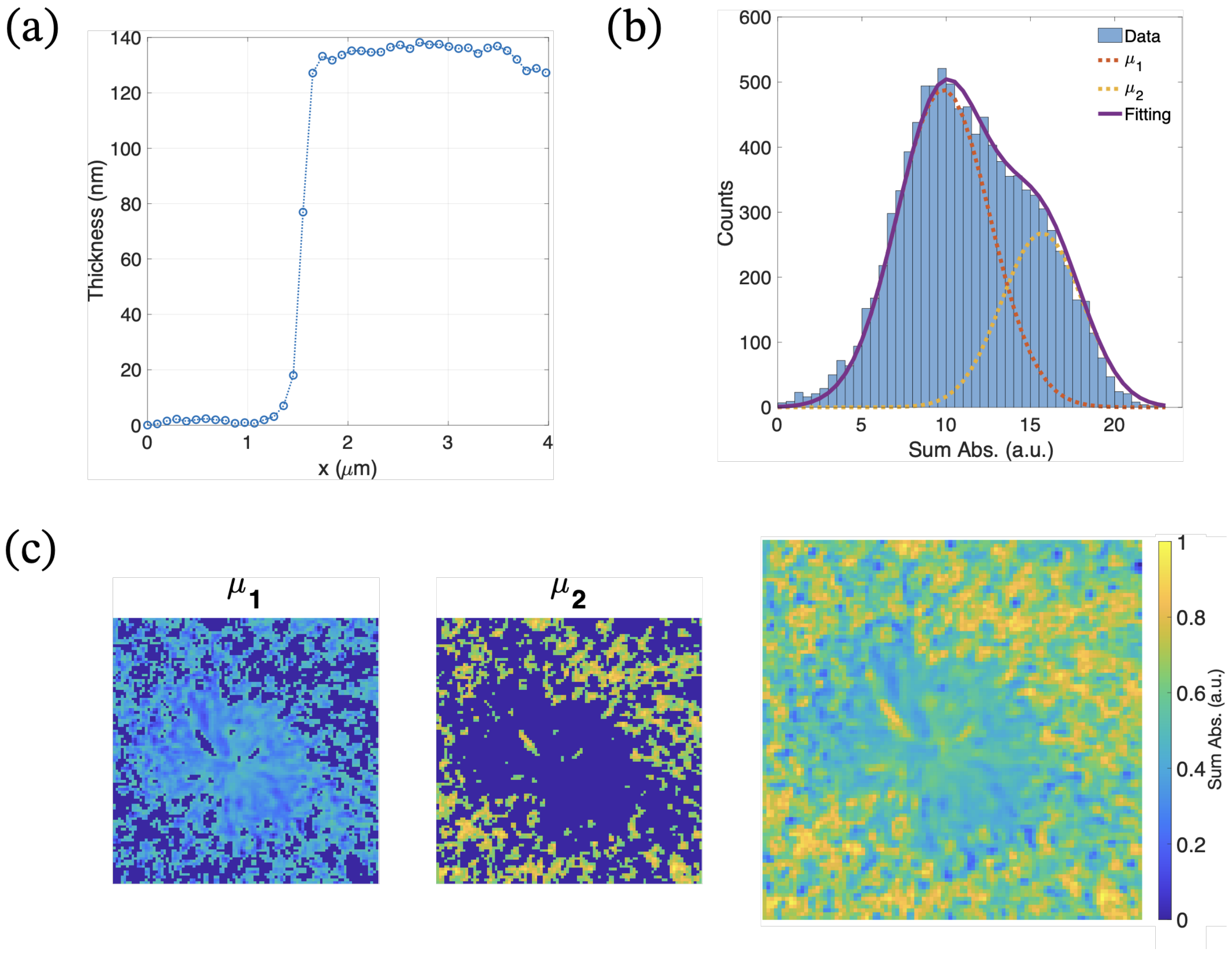

Figure S1: (a) AFM determined thickness characterization from the edge of the film towards the covered area. (b) A histogram of the sum of absorption and (c) the absorption map (right) and the distributions of the components corresponding to μ_1 (left), μ_2 (center).

Power Dependent Photoluminescence

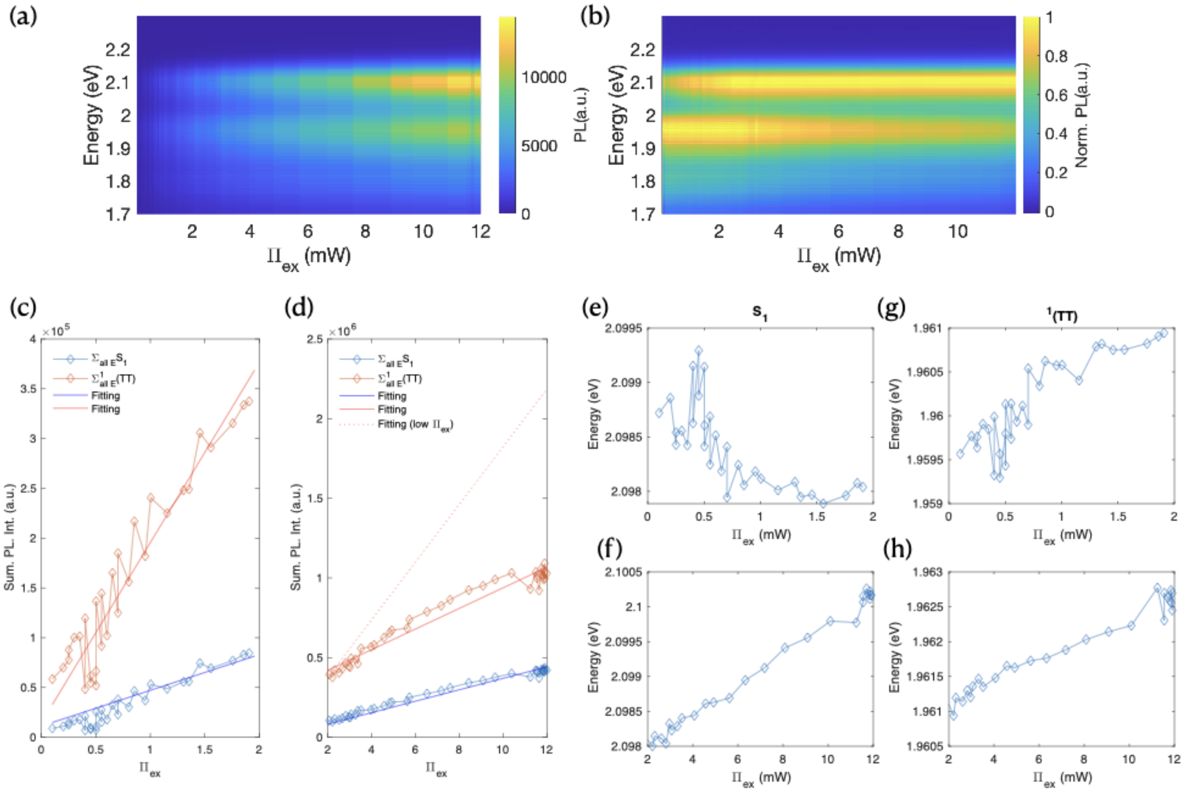

Figure S2: (a) Power-dependent photoluminescence (PL) at 150 K. A 405 nm continuous wave (CW) laser was used to excite the sample. (b) Normalized power-dependent PL (c) The sum of emission of S_1 and $^1(TT)$ with low excitation power (below 2 mW) (d) The sum of emission of of S_1 and $^1(TT)$ with high excitation power (above 2 mW) (e) The emission energy of S_1 with low excitation power (below 2 mW) and high excitation power (above 2 mW). (g) The emission energy of $^1(TT)$ with low power excitation (below 2 mW) and (h) high excitation power

At 150 K, steady-state PL measurements of diF-TES-ADT reveal that S_1 emission exhibits stronger excitation density dependence than the two $^1(TT)$ -related peaks (Fig. S2). The prompt S_1 emission follows monomolecular recombination, scaling linearly with exciton density. In contrast, the $^1(TT)$ population arises from both prompt transitions from the biexcitonic state and delayed backward TTA from T_1, with the latter scaling quadratically

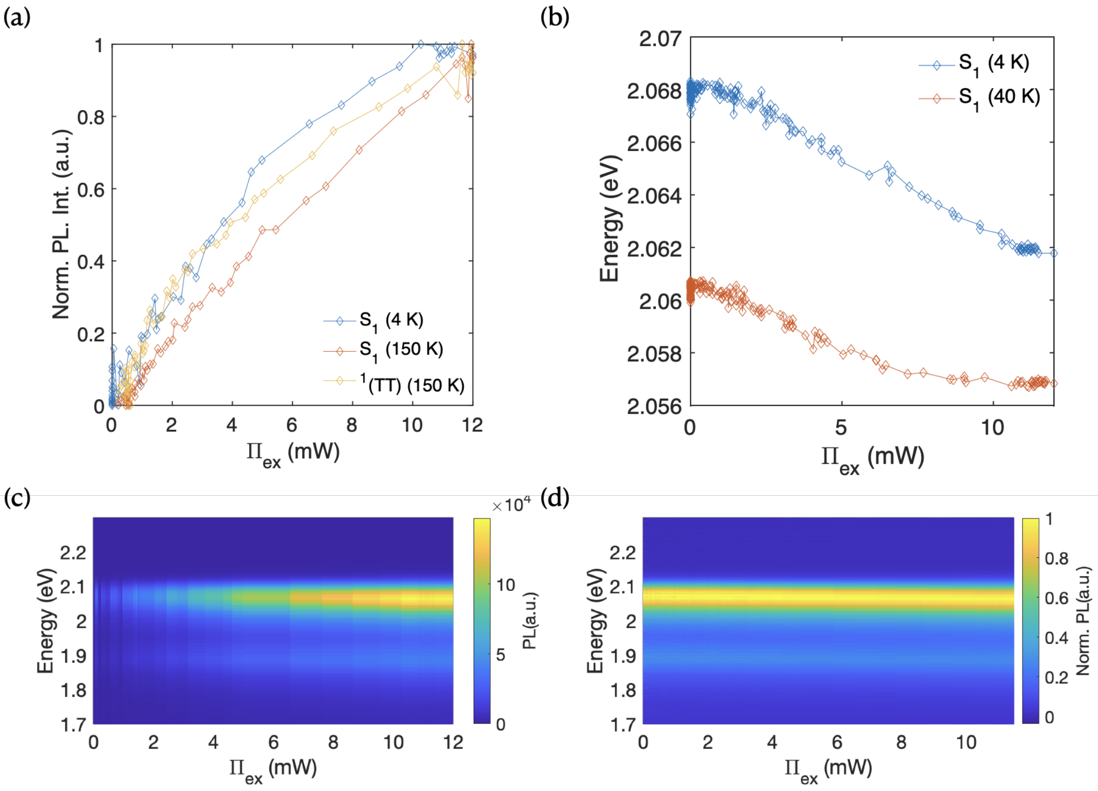

Figure S3: (a) Power dependent S_1 intensity at 4 K, and S_1 and $^1(TT)$ at 150 K. (b) The power dependent S_1 energy shift at 4 K and 40 K. (c) Power dependent PL at 4 K, (d) Normalized power dependent PL at 4 K.

with triplet density.[S1,S2]

The $^1(TT)$ state exhibits two distinct regimes in both recombination rate and energy shift. At low excitation powers ($\Pi_{ex} < 2.0\,\mathrm{mW}$, corresponding to 4.0×10^{18} photons $\mathrm{s^{-1}\,cm^{-2}}$), the $^1(TT)$ emission intensity increases more rapidly than at high powers ($\Pi_{ex} > 2.0\,\mathrm{mW}$). The ratio of $^1(TT)$ to S_{0-0} emission gradients ($dI_{\mathrm{TT}}/dI_{\mathrm{S}}$) decreases from 5.0 to 1.8 between these regimes.

While time-resolved measurements indicate that emission enhancement primarily stems from TTA, under continuous excitation the long-lived states ($^1(TT)$, $(T..T)^l$, and T_1) dominate the steady-state PL.[S1] The $^1(TT)$ state continues to populate from S_1 , as backward TTA occurs on microsecond timescales compared to prompt SF within 100 ps.[S2,S3] At high excitation densities, $^1(TT)$ saturation effectively reduces TTA, leading to the observed gradient approaching unity.[S1] The enhanced geminate recombination rates of $^1(TT)$ in the low-power regime align with efficient SF and TTA processes in diF-TES-ADT .[S1]

Stokes Shift

The local Stokes shift can be calculated at each point in the map as shown in Fig S5, showing a weak but significant negative correlation with η_{SF} of r=-0.023. That supports local energy alignment of the primary transition (S_{0-0}) given by the monomolecular properties which is less relevant to intermolecular states relevant to the SF process.[S4,S5]

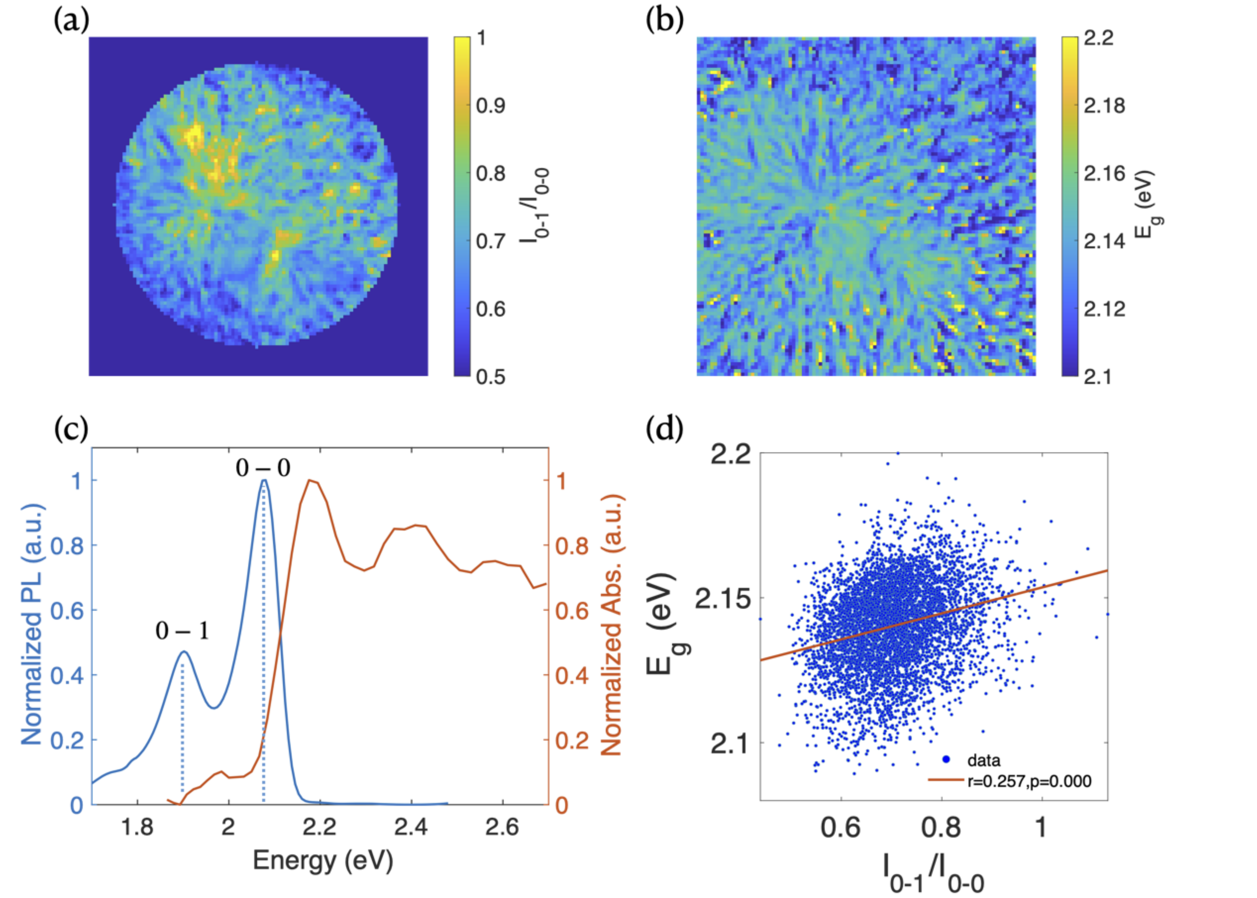

Figure S4: (a) Relative intensity map between 2 peaks: I_{0-1}/I_{0-0}. (b) The absorption edge energy map of diF-TES-ADT at 4 K. (c) Average PL and absorption of diF-TES-ADT film at 4 K. (d) The correlation between the I_{0-1}/I_{0-0} and absorption edge energy.

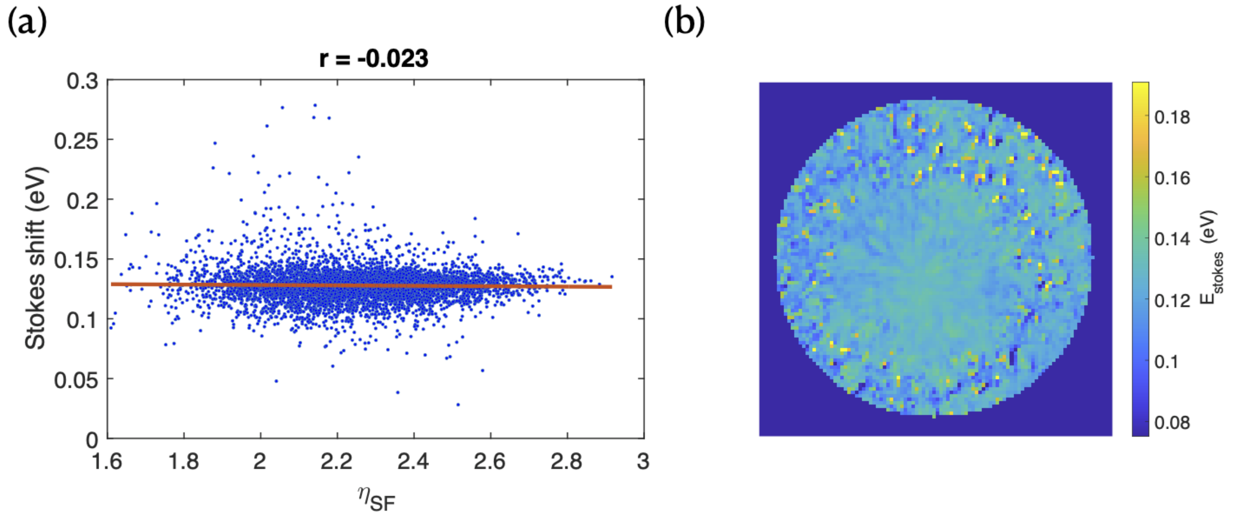

Figure S5: (a) The correlation between the local Stokes shift energy and SF efficiency at 150 K. (b) The map of Stokes shift at 150 K.

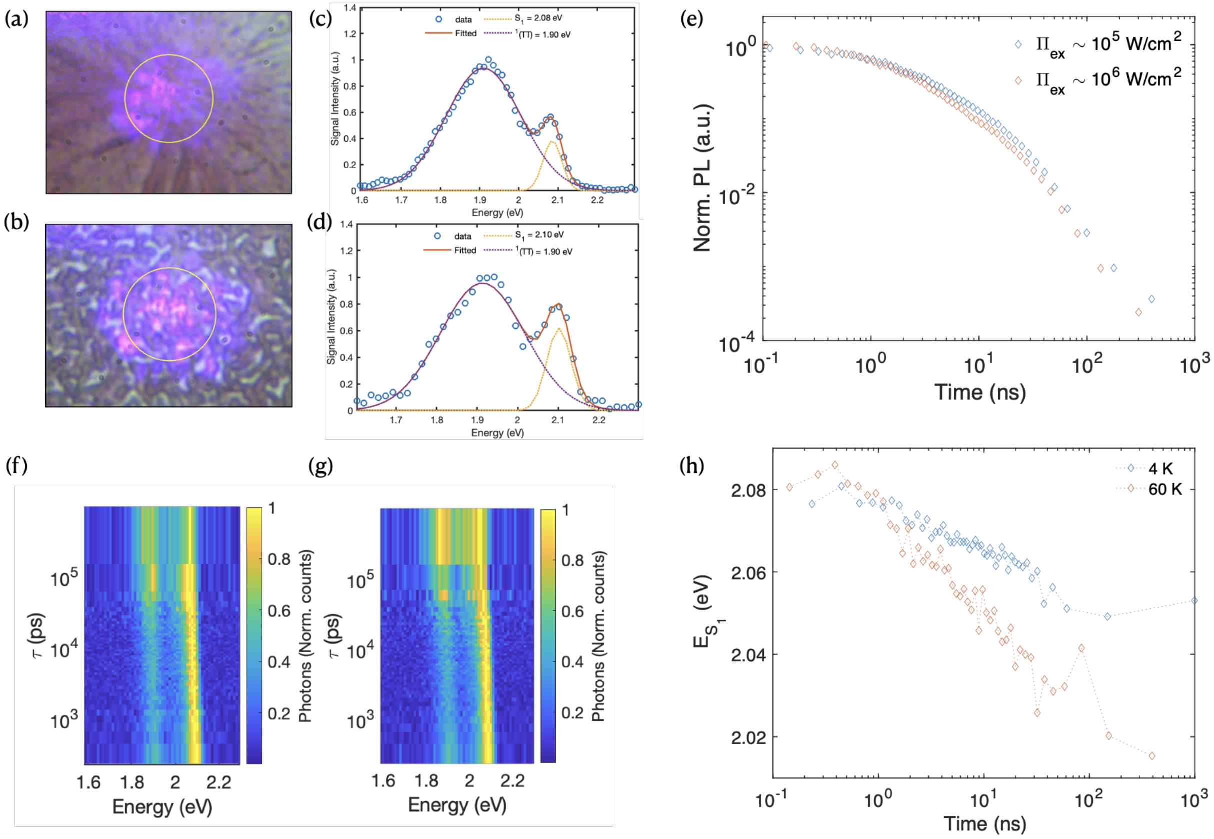

Figure S6: Optical image of the (a) high crystalline spot of diF-TES-ADT film and (b) low crystalline spot. (c-d) The corresponding PL spectrum to the selected part of film (a) and (b) integrated over time. (e) Power dependent $^1(TT)$ lifetime measurement. (f-g) Time resolved spectral evolution of diF-TES-ADT film at 4 K in (f) high crystalline region and (g) poor crystalline region. (h) Low temperature (4 K and 60 K) S_1 lifetime measurements.

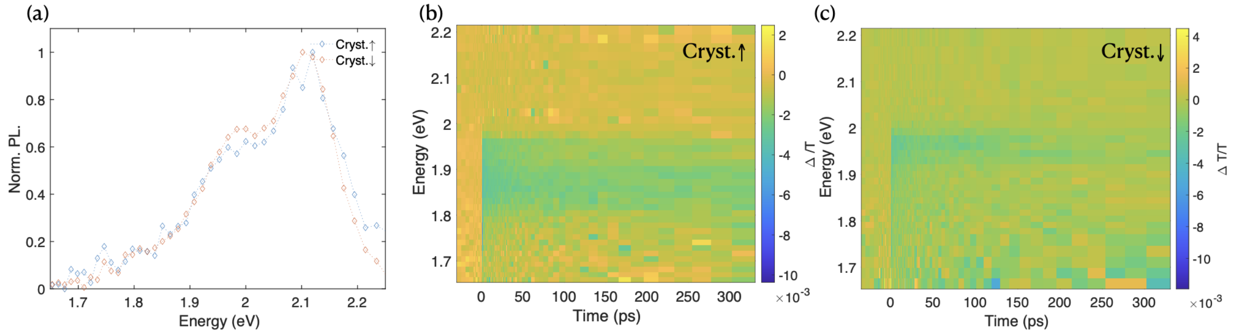

Figure S7: (a) The PL spectrum of highly and lower crystalline regions. The full spectral evolution of $\Delta T/T$ for 300 ps in (b) high crystalline and (c) lower crystalline regions.

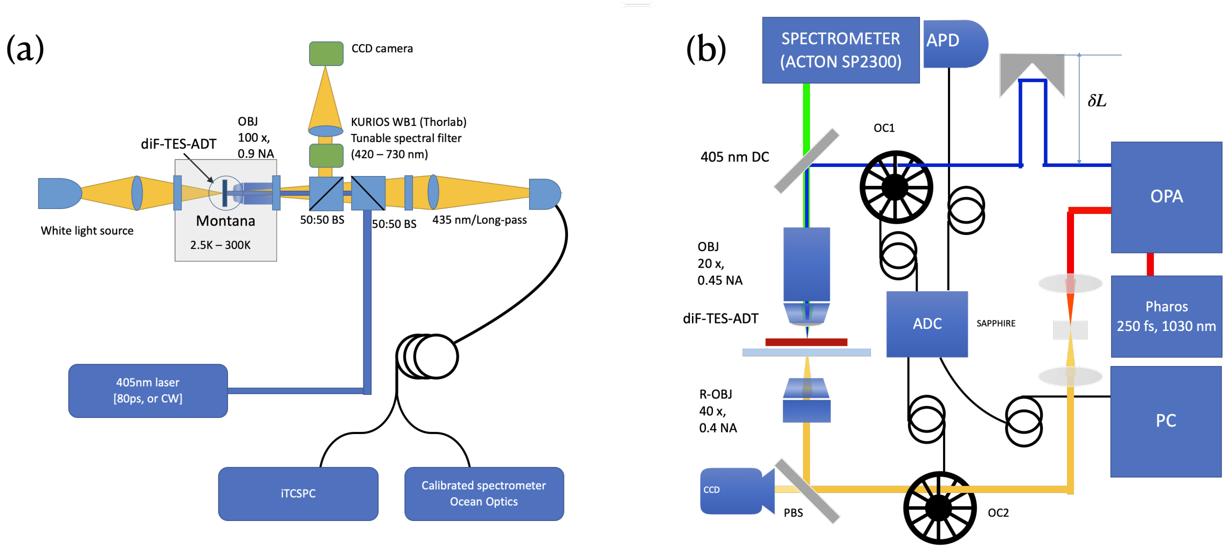

Figure S8: (a) The layout of the hyperspectral image processing setup. BS is the beam-splitter, and OBJ is the objective lens. (b) The layout of μ-TA setup. ADC is the analogue-to-digital converter. OPA is optical parametric amplifier, and APD is an avalanche photo diode.

References

(S1) Bossanyi, D. G.; Matthiesen, M.; Wang, S.; Smith, J. A.; Kilbride, R. C.; Shipp, J. D.; Chekulaev, D.; Holland, E.; Anthony, J. E.; Zaumseil, J., et al. Emissive spin-0 triplet-pairs are a direct product of triplet–triplet annihilation in pentacene single crystals and anthradithiophene films. *Nature chemistry* **2021**, *13*, 163–171.

(S2) Yong, C. K.; Musser, A. J.; Bayliss, S. L.; Lukman, S.; Tamura, H.; Bubnova, O.; Hallani, R. K.; Meneau, A.; Resel, R.; Maruyama, M., et al. The entangled triplet pair state in acene and heteroacene materials. *Nature communications* **2017**, *8*, 1–12.

(S3) Mayonado, G.; Vogt, K. T.; Van Schenck, J. D.; Ostroverkhova, O.; Graham, M. W. Packing Morphology-Dependent Singlet Fission in Single Crystal Anthradithiophene Derivatives. International Conference on Ultrafast Phenomena. 2020; pp Th2A–4.

(S4) Morrison, A. F.; Herbert, J. M. Evidence for singlet fission driven by vibronic coherence in crystalline tetracene. *The Journal of Physical Chemistry Letters* **2017**, *8*, 1442–1448.

(S5) Mayonado, G.; Vogt, K. T.; Van Schenck, J. D.; Zhu, L.; Fregoso, G.; Anthony, J.; Ostroverkhova, O.; Graham, M. W. High-Symmetry Anthradithiophene Molecular Packing Motifs Promote Thermally Activated Singlet Fission. *The Journal of Physical Chemistry C* **2022**, *126*, 4433–4445.
